# Outcomes of Liver Transplantation in Incidental Intrahepatic Cholangiocarcinoma and Combined Hepatocellular-Cholangiocarcinoma: An Exceptional Perspective from a Single-Center Experience

**DOI:** 10.3390/jcm14248857

**Published:** 2025-12-15

**Authors:** Lijie Ma, Qiang Xia, Meng Sha

**Affiliations:** 1 Department of Liver Surgery, Renji Hospital, School of Medicine, Shanghai Jiao Tong University, Shanghai 200127, China; 15821523323@163.com; 2 Shanghai Institute of Transplantation, Shanghai 200127, China; 3 Shanghai Engineering Research Centre of Transplantation and Immunology, Shanghai 200127, China

**Keywords:** liver transplantation, incidental, intrahepatic cholangiocarcinoma, combined hepatocellular cholangiocarcinoma, outcome

## Abstract

**Background/Objectives**: Intrahepatic cholangiocarcinoma (ICC) and combined hepatocellular-cholangiocarcinoma (CHC) have historically been considered a contraindication for liver transplantation (LT) due to poor prognosis. However, incidental ICC/CHC has been reported in small amounts of patients undergoing LT. **Methods**: A retrospective cohort study was conducted to analyze patients undergoing LT with incidental ICC/CHC at our center between January 2010 and December 2021. **Results**: 28 patients including 12 incidental ICCs and 16 CHCs on explant were identified. Median follow-up after LT was 63 months and 13 patients died due to tumor recurrence. The 1-, 3-, and 5-year survival rates for the whole group were 85.7%, 64.3%, and 53.3%, respectively. There was no significant difference in survival rates between the ICC and CHC groups. RFS and OS in the group with tumors less than 3 cm at 1, 3, and 5 years were 85.7%, 78.6%, and 70.7% and 92.9%, 78.6%, and 64.3%, respectively, which were significantly higher than those with tumors over 3 cm (*p* = 0.029 and 0.089, respectively). Additionally, patients within the Milan criteria also had a superior RFS (*p* = 0.032) and OS trend (*p* = 0.097) when compared with those beyond the Milan criteria. **Conclusions**: These results suggest that LT could be an option for highly selected patients with an early stage of ICC/CHC.

## 1. Introduction

Intrahepatic cholangiocarcinoma (ICC) is the second most common primary liver tumor, with increasing incidence rate worldwide in recent years [1]. Due to advanced tumor and distant metastasis at early stage, more than 50% of patients lose opportunities for radical surgery [2]. As a result, the 5-year overall survival rate is barely at 30% despite improvements in chemotherapeutic drugs and surgical techniques [3,4]. Combined hepatocellular-cholangiocarcinoma (CHC) is a less-frequent liver malignancy and accounts for 5% of all primary liver carcinoma [5]. This kind of tumor has both hepatocytic and cholangiocytic differentiation within the same lesion, which implies a worse biological behavior and poorer prognosis [6,7].

Since radical resection is the only way to achieve long-term survival, liver transplantation (LT) has emerged as a potential curative option, offering a total hepatectomy and tumor removal. Unfortunately, early experience of LT for unselected ICC and CHC showed high recurrence rates and a disappointing prognosis, with 5-year survival rates of less than 25% [8,9]. However, recent accumulating data suggest that highly selected patients may also benefit from LT [10]. Sapisochin et al. showed that post-LT OS for “very early” ICC patients (single tumor ≤ 2 cm) were 100%, 73%, and 73% at 1, 3, and 5 years [11]. Using the protocol of neoadjuvant therapy for locally advanced unresectable ICC, consisting of gemcitabine-based chemotherapy, such as gemcitabine-cisplatin or gemcitabine-capecitabine, with second-line or third-line therapies given per institutional standards, the post-LT OS were 100%, 83.3%, and 83.3% at 1, 3, and 5 years [12]. Retrospective analysis for CHC also revealed that LT could provide superior survival compared to liver resection in the setting of cirrhosis and tumors smaller than 5 cm [13].

Currently, the standard diagnostic and therapeutic approach for hepatic malignancies is preoperative alpha-fetoprotein (AFP) and carcinoembryonic antigen19-9 (CA19-9) combined with CT, MRI, and PET-CT. However, some patients, particularly those with cirrhosis, exhibit neither typical imaging features nor markedly elevated tumor markers, ultimately posing challenges to the accurate diagnosis of ICC and CHC [14,15]. Consequently, the incidental detection of previously undetected or misdiagnosed ICC and CHC is frequently observed in LT recipients [16]. Against this backdrop, our study aims to analyze the post-transplant prognosis of patients with incidental ICC and CHC at our center, while also endeavoring to identify potential prognostic factors in this patient group.

## 2. Materials and Methods

### 2.1. Study Design

A retrospective cohort study including patients undergoing LT with incidental pathological diagnosis of ICC/CHC at our center between January 2010 and December 2021 was conducted. The incidental ICC/CHC was defined as previously undetected tumors or misdiagnosed as HCC on preoperative enhanced CT (Siemens Definition AS 64-slice 128-layer spiral CT scanner) and MRI (Siemens Superconducting MAGNETOM Avanto 1.5T Magnetic Resonance Imaging System, XA60), but confirmed as ICC or CHC on histopathological examinations of the explanted liver.

The research described in this manuscript involving clinical data collection and analysis is approved by the Ethical Committee’s Institutional Review Board of Renji Hospital (KY2022-117-B), Shanghai Jiao Tong University School of Medicine. All participants and their authorized agents were told and had signed informed consent that their medical history and follow-up data might be used for academic analysis before being involved in our follow-up system. None of the organs were procured from executed prisoners and the organs were procured after informed consent with the full record in the China Organ Transplant Response System (https://www.cot.org.cn/ accessed on 31 July 2013, and https://cltr.cotr.cn/, accessed on 28 February 2005). The study was conducted according to the guidelines of the Declaration of Helsinki.

### 2.2. Data Collection and Follow-Up

Pretransplant patient data including age, gender, underlying liver disease, infection of hepatitis virus, serum AFP level, CA19-9 level, and preoperative treatment were collected. Data of cirrhosis, tumor number, maximum tumor size, grade of differentiation, presence of vascular or perineural invasion, lymph node metastasis, and tumor stage were confirmed through postoperative pathology.

The pathological assessment of the surgical specimen following LT was conducted by two senior pathologists who specialize in hepatic tumors. Routine immunohistochemical staining was performed concurrently with the H&E slide evaluation, including AFP, arginase, HepPar-1, CD34, CK, GPC-3, EpCAM, and HSP70, among others.

All patients received orthotopic liver transplantation. Postoperative immunosuppressive treatment included a regimen consisting of tacrolimus, mycophenolate mofetil (MMF), and steroids. After 1 month post-LT, steroids were withdrawn. MMF was switched to sirolimus 3 months after LT combined with a low level of tacrolimus.

During the follow-up, liver function, serum AFP, CA19-9 level, and ultrasound were monitored every month. To allow early detection of tumor recurrence, CT scans of the chest and abdomen were performed every 3 months during the first year and every 6 months thereafter. If tumor recurrence was confirmed, the optimal treatment was provided individually for each patient. Data of tumor recurrence, mortality and cause of death were collected for all included patients.

### 2.3. Statistical Analysis

Continuous variables were described using medians with ranges and categorical variables are expressed as numbers with ratio. A Chi-square test, Student’s *t*-test, or Mann–Whitney U test was used to compare continuous and categorical variables, respectively. Analysis of overall survival (OS) and recurrence-free survival (RFS) were performed using Kaplan–Meier analysis and compared with the log-rank test. The statistical analysis was performed using SPSS Statistics, version 25 (SPSS, INC., Chicago, IL, USA). A *p*-value of <0.05 was considered statistically significant.

## 3. Results

### 3.1. Patient Characteristics

A total of 28 patients with incidental ICC/CHC on explant were identified at our center between January 2010 and December 2021. The median follow-up after LT was 63 months. 13 patients died from tumor recurrence; 1 patient died due to COVID-19 without tumor recurrence. The patient characteristics were summarized in Table 1. Among them, 25 patients were presumed as HCC preoperatively and 3 patients were incidentally found to have tumor lesions on the explanted liver. The median age was 52 years (range, 35–73 years). Most patients were male (82.1%) and HBV infection (78.6%) was the most common etiology of cirrhosis. The majority of patients were within the Milan criteria (71.4%) with a single nodule (71.4%) and median maximum size of 3.3 cm. Well differentiation of the tumor was found in 16 patients (57.1%) and presence of micro-vascular invasion was present in 11 patients (39.3%).

### 3.2. Analysis Based on Tumor Types

**Figure 1:**
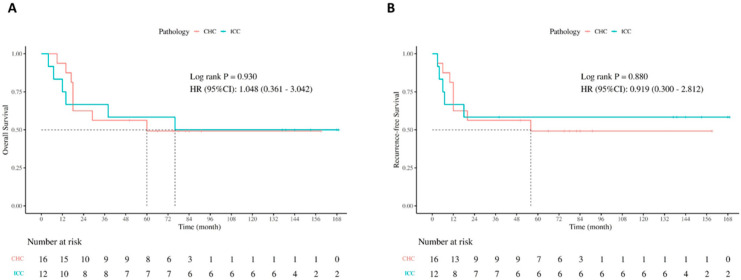
Survival comparison by tumor pathology type: (**A**) OS in patients with CHC versus ICC, (**B**) RFS in patients with CHC versus ICC.

Pathological examination confirmed incidental ICC in 12 patients and CHC in another 16 patients. Tumors that were previously undetected were found in 3 patients in the ICC group, while 16 patients in the CHC group were all presumed as HCC on preoperative imaging. The level of serum CA19-9 was higher in the ICC group compared with the CHC group (*p* = 0.007). The other variables including gender, HBV infection, presence of cirrhosis, AFP level, maximum tumor size, and tumor grade did not differ between the two groups. Comparisons of OS and RFS in each tumor type are shown in Table 2 and Figure 1. The 1-, 3-, and 5-year survival rates were 75%, 66.7%, and 58.3% for ICC and 93.8%, 62.5%, and 49.2% for CHC, respectively. No significant difference was observed between the ICC and CHC groups in terms of OS and RFS (*p* = 0.930 and 0.880, respectively).

### 3.3. Analysis Based on Tumor Size

**Figure 2:**
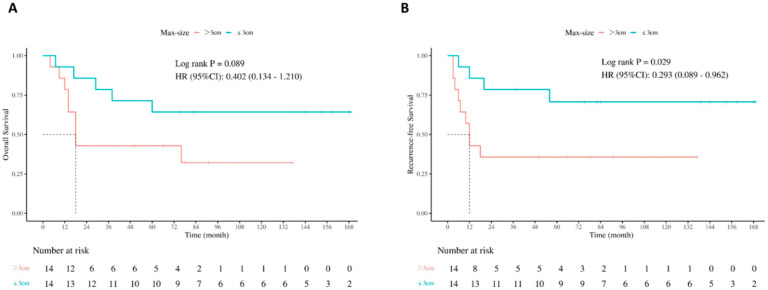
Survival comparison by tumor size: (**A**) OS in patients with tumor over 3 cm versus less than 3 cm, (**B**) RFS in patients with tumor over 3 cm versus less than 3 cm.

Patients were further classified according to the maximum tumor size. 12 patients with ICC were divided into >3 cm (n = 5) and ≤3 cm (n = 7), while 16 CHC patients were divided into >3 cm (n = 9) and ≤3 cm (n = 7) (Table 3). Patients in the ≤3 cm group of ICC and CHC were both within the Milan criteria, which was higher than those in the >3 cm group. Comparisons of other variables did not show significant differences between groups. We next compared the survival rates. As shown in Figure 2A,B, in all patients, individuals with tumors ≤3 cm had significantly better RFS (*p* = 0.029) and a superior OS trend (*p* = 0.089) when compared with patients with tumors >3 cm. In subgroup analysis by tumor size in each tumor type, a numerical trend toward improved RFS was observed in patients with tumors ≤3 cm than those with tumors >3 cm (Supplementary Figure S1A,B). In CHC patients, a similar superior survival trend was presented in the ≤3 cm group, though no statistical difference was reached (RFS, *p* = 0.359; OS, *p* = 0.455) (Supplementary Figure S1C,D).

### 3.4. Analysis Based on Milan Criteria

**Figure 3:**
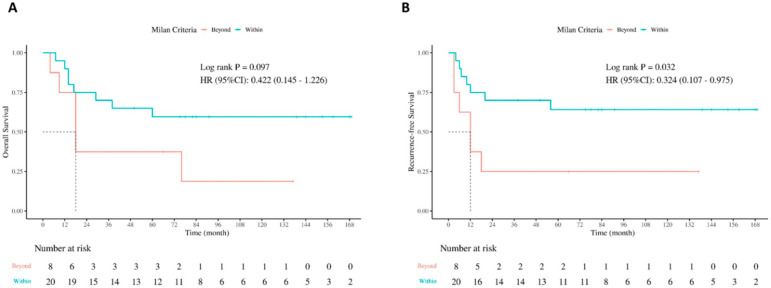
Survival comparison by Milan criteria: (**A**) OS in patients within Milan criteria versus beyond Milan criteria, (**B**) RFS in patients within Milan criteria versus beyond Milan criteria.

To explore the impact of selecting criteria, outcomes of patients were compared based on the Milan criteria. As shown in Table 4, 9 ICC patients and 11 CHC patients within the Milan criteria had significantly smaller tumor size than those beyond the Milan criteria. Further survival analysis showed that patients within the Milan criteria had better RFS (*p* = 0.032) and OS (*p* = 0.097) when compared with patients beyond the Milan criteria (Figure 3A,B). In subgroup analysis stratified by tumor type, no significant difference in RFS and OS were found in ICC patients according to Milan criteria (Supplementary Figure S2A,B), while in patients with CHC, superior survival rates were revealed in patients within the Milan criteria (RFS, *p* = 0.038; OS, *p* = 0.087) (Supplementary Figure S2C,D).

## 4. Discussion

Liver transplantation for ICC/CHC has long been debated due to previous high recurrence and poor survival rate. Our present study analyzed the outcomes of LT in incidental ICC/CHC after pathological examination based on a single-center series. A total of 28 patients, including 12 diagnosed as ICC and 16 diagnosed as CHC, were included in the study. After a median follow-up of 63 months, the 1-, 3-, and 5-year survival rates for the whole group were 85.7%, 64.3%, and 53.3%, respectively, which were aligned with those of 82.1%, 68.7%, and 57.1% in Parker R’s cohort [16]; 92.3%, 76.9%, and 76.9% in Óscar Caso-Maestro’s cohort [17]; 79%, 63%, and 46% in Ohdan H’s cohort [18]; and 83%, 70%, and 60% in Charco R’s cohort [19].

**Figure 4:**
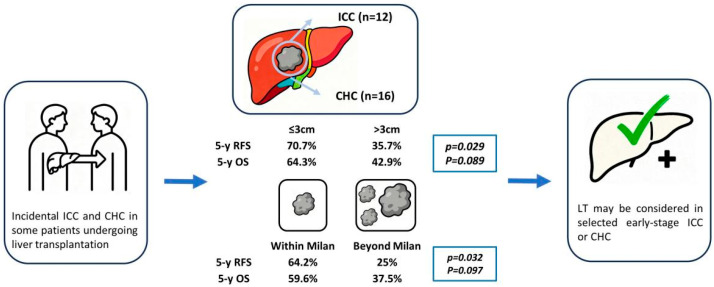
Overview of outcomes after liver transplantation with incidental ICC/CHC.

In the analysis stratified by tumor type, there was no significant difference in terms of OS and RFS between the ICC and CHC groups. Further, patients were divided into two groups according to tumor size. In our series, 14 patients had tumors ≤3 cm and this group of patients showed 5-year survival rates of 64.3%, which was significantly higher than those with tumors >3 cm. Additionally, patients within the Milan criteria had a superior RFS and OS trend when compared with those beyond the Milan criteria (Figure 4). The results are consistent with previous studies which determined tumor size as the main criteria [20]. Sapisochin et al. first defined a single tumor less than 2 cm as very early ICC [11]. In the multicenter international analysis, the 5-year survival rate was 65% in the very-early group compared with 45% in the other patients [21]. The cumulative recurrence risk was only 18% in the very-early ICC group. Recently, De Martin et al. also compared LT and liver resection for patients with ICC/CHC less than 5 cm (all in cirrhosis), reporting a better 5-year OS rate of 65% by LT [13]. These findings suggest that LT may provide a favorable prognosis in patients with early stage of ICC/CHC.

However, it is noted that preoperative tumor detection for small-size ICC/CHC less than 3 cm by imaging can be difficult. Generally, cholangiocarcinoma is a hypo-vascular tumor with peripheral rim-like enhancement during the arterial phase through CT or MRI [22]. However, in the cases of liver cirrhosis or chronic viral hepatitis, a proportion of cholangiocarcinoma appears as hyper-vascular tumors, similar to HCC [23,24]. Therefore, precise differential diagnosis between HCC and ICC/CHC through imaging is challenging. On the other hand, different tumor markers may be helpful to distinguish tumor types, though not always accurate. An increased level of AFP is generally recognized as one of the markers for HCC development [25], while CA19-9 is more associated with cholangiocarcinoma [26]. In the present study, incidental CHC with both hepatocytic and cholangiocytic differentiation showed elevated AFP but a normal level of CA19-9. This result may contribute to the preoperative misdiagnosis of these patients as HCC. Under this circumstance, liver biopsy may be recommended for small nodules with atypical imaging features and tumor markers [27]. However, it is noted that biopsy should be performed with caution in patients with poor coagulation function or ascites.

ICC has an insidious presentation and a high postoperative recurrence rate. Traditionally, only 20–30% of ICC patients qualify for curative resection [2]. Recent evidence revealed that tumor behavior and response to neoadjuvant therapy poses another important factor for patient selection despite tumor size [28]. In a prospective study, a protocol of neoadjuvant therapy was developed for locally advanced unresectable ICC. Patients who demonstrated disease stability for 6 months were eligible for LT [12]. The 5-year survival for these patients was 83.3% with a recurrence rate of 50%, suggesting that sustained response to neoadjuvant therapy could serve as a surrogate marker of tumor biology rather than tumor size [29,30]. Similar results were also reported by the UCLA group through a three-decade analysis [31]. These findings appear to be a better strategy to select cholangiocarcinoma patients for LT and may greatly expand the potential recipients. In our study, however, it is interesting to find that patients who received preoperative treatment showed worse outcomes than those who did not (Supplementary Figure S3A,B). Due to the incidental nature of these tumors on explant, the selected preoperative treatment therapy, such as transcatheter arterial chemoembolization (TACE) and radiofrequency ablation (RFA), may be ineffective against ICC/CHC, which consequently leads to adverse outcomes and poor prognosis [32,33]. At the molecular level, around 25% of tumors harbor IDH1/2 mutations and 10–15% exhibit FGFR2 fusions [34]. The corresponding targeted therapy, Pemigatinib, has received FDA approval for previously treated, locally advanced or metastatic cholangiocarcinoma [35]. Alterations in *KRAS*, *TP53,* and *CDKN2A*, among others, indicate a high risk of recurrence. Moreover, the multi-omics atlas further subdivides ICC into inflammatory, proliferative, and metabolic subtypes [36], providing a basis for preoperative risk stratification and postoperative adjuvant therapy. In addition, the treatment regimens utilized in our cohort were diverse and inconsistent, making it difficult to evaluate the efficacy and response to preoperative treatment. Therefore, it emphasizes the importance of clarifying the tumor pathological type preoperatively and formulating a standardized downstaging regimen.

Living donor liver transplantation (LDLT) provides a timely opportunity for curative treatment for patients with ICC amid organ shortages [37]. For cases that are unresectable but have downstaged tumors following neoadjuvant therapy and favorable tumor biology, LDLT can avoid lengthy waiting times, reduce withdrawal rates, achieve R0 resection, and address insufficient residual liver volume [38,39]. This approach is particularly viable in regions with low donor availability, as it provides a practical way to expand the criteria for liver transplantation for ICC patients.

The early switch to a Sirolimus (SRL)-based regimen is a critical detail, given its potential anti-neoplastic properties, and may represent one potential factor contributing to our study’s superior results compared to previous investigations. A triple sequential regimen involving the discontinuation of glucocorticoids in the first month, the replacement of mycophenolate mofetil with SRL after three months, and the maintenance of a low tacrolimus (Tac) dosage has been shown to balance anti-rejection efficacy with reduced drug toxicity in multiple clinical studies, and was applied to all patients uniformly in this study. The core evidence and key points are as follows: (1) Rapid steroid withdrawal significantly reduces metabolic complications such as hypertension, new-onset diabetes, and osteoporosis without increasing the risk of acute rejection. (2) Switching from mycophenolate mofetil (MMF) to SRL around three months post-transplant while continuing low-dose Tac leverages a complementary mechanism—Tac inhibits initial T-cell activation, and SRL blocks IL-2 signaling. This maintains potent immunosuppression while reducing calcineurin inhibitor (CNI) nephrotoxicity and cardiovascular risks. (3) A significant reduction in leukopenia, bacterial infections, and metabolic side effects was observed. However, large-scale, long-term, randomized controlled trials are still required to validate the impact of this approach on chronic rejection, graft fibrosis and tumor recurrence. Furthermore, the target trough concentration for SRL (multicenter recommendations suggest 4–8 ng/mL) and the minimum effective exposure for Tac (C0 3–5 ng/mL) must be established.

The present study had several limitations. First, this was a single-center retrospective study with a limited number of patients, while subgroup analyses were underpowered. Meanwhile, the small sample size prevents the identification of independent prognostic factors through the multivariate analysis. Second, due to the wide time span of follow-up, several factors including surgical advancements, imaging evaluations, and perioperative treatment regimens may change over time, which limited the consistent analysis. Additionally, detailed information regarding adjuvant therapy, treatment for recurrence, and other interventions was unclear and incomplete. Despite the limitations above, this is the first report from China describing outcomes of LT for patients with incidental ICC/CHC. In contrast to previous studies, the analysis was also stratified by different tumor types, tumor size, and selection criteria to identify potential factors impacting outcomes. Survival rates after LT for recipients with incidental ICC/CHC are acceptable. Patients with early stages of ICC/CHC had better survival and may benefit from LT. Future multicenter prospective studies are expected to confirm the results and establish appropriate selection criteria for patients with ICC/CHC awaiting LT.

## 5. Conclusions

In highly selected patients with incidental ICC/CHC, particularly those with smaller tumor burdens (e.g., within the Milan criteria or with tumors ≤3 cm), liver transplantation can achieve acceptable long-term survival. These findings support further prospective evaluation of selection criteria for these diagnoses.

## Figures and Tables

**Table 1:** Overview of patient demographics and tumor pathological characteristics.

Variables	Total (n = 28)	CHC (n = 16)	ICC (n = 12)	*p*-Value
Age	52 (35–73)	53 (35–62)	51 (39–73)	0.625
Gender, Male	23 (82.1)	13 (81.3)	10 (83.3)	1.000
Presence of HBV infection	22 (78.6)	11 (68.8)	11 (91.7)	0.196
Presence of cirrhosis	21 (75.0)	12 (75.0)	9(75.0)	1.000
AFP level (ng/mL)	107.4 (4.1–9950)	193.0 (4.1–9950)	25.5 (4.8–3000)	0.302
CA19-9 level (U/mL)	25.2 (2.0–325)	18.9 (2–82)	42.3 (14.5–325)	0.007
Maximum tumor size (cm)	3.3 (0.8–12.0)	3.5 (1.5–10.0)	3 (0.8–12.0)	
Tumor number, Single	20 (71.4)	12 (75)	8 (66.7)	0.691
Presence of micro-vascular invasion	11 (39.3)	7 (43.8)	4 (33.3)	0.705
Differentiation				0.242
Well	16 (57.1)	11 (68.8)	5 (41.7)	
Moderate	8 (28.6)	4 (25.0)	4 (33.3)	
Poor	4 (14.3)	1 (6.2)	3 (25.0)	
Presence of perineural invasion	2 (7.1)	1 (6.3)	1 (8.3)	1.000
Within Milan criteria	20 (71.4)	11 (68.8)	9 (75.0)	1.000
Tumor stage				
I	12 (42.9)	7 (43.8)	5 (41.7)	0.680
II	15 (53.6)	9 (56.2)	6 (50.0)	
III	1 (3.5)	0	1 (8.3)	
Type				
Incidental tumor	3 (10.7)	0	3 (25.0)	0.067
Presumed HCC	25 (89.3)	16 (100.0)	9 (75.0)	
Preoperative treatment	10 (35.7)	7 (43.8)	3 (25.0)	0.434
Postoperative adjuvant therapy	5 (17.9)	3 (18.8)	2 (16.8)	1.000

Continuous variables were expressed as median and range. Categorical variables were expressed as frequencies (n, %). Abbreviations: CHC, combined hepatocellular-cholangiocarcinoma; ICC, intrahepatic cholangiocarcinoma; HCC, hepatocellular carcinoma; HBV, hepatitis B virus; AFP, alpha-fetoprotein; CA19-9, carcinoembryonic antigen19-9.

**Table 2:** Survival comparisons by tumor type, maximum tumor size, and selection criteria.

	ICC			CHC			*p*-Value
	1-Year	3-Year	5-Year	1-Year	3-Year	5-Year	
RFS%	66.7	58.3	58.3	62.5	56.3	49.2	0.880
OS%	75	66.7	58.3	93.8	62.5	49.2	0.930
	<3 cm			≥3 cm			*p*-value
	1-year	3-year	5-year	1-year	3-year	5-year	
RFS%	85.7	78.6	70.7	42.9	35.7	35.7	0.029
OS%	92.9	78.6	64.3	78.6	42.9	42.9	0.089
	Within Milan criteria			Beyond Milan criteria			*p*-value
	1-year	3-year	5-year	1-year	3-year	5-year	
RFS%	75	70	64.2	37.5	25	25	0.032
OS%	90	75	59.6	75	37.5	37.5	0.097

**Table 3:** Comparisons according to tumor size in subgroups.

	ICC (n = 12)			CHC (n = 16)		
Variable	Size > 3 cm (n = 5)	Size ≤ 3 cm (n = 7)	*p*-Value	Size > 3 cm (n = 9)	Size ≤ 3 cm (n = 7)	*p*-Value
Age, years	50 (49–58)	52 (39–73)	1.000	52 (35–62)	54 (46–61)	0.490
Gender, Male	4 (80.0)	6 (85.7)	1.000	6 (66.7)	7 (100.0)	0.212
Presence of HBV infection	5 (100.0)	6 (85.7)	1.000	6 (66.7)	5 (71.4)	1.000
Presence of cirrhosis	4 (80.0)	5 (71.4)	1.000	6 (66.7)	6 (85.7)	0.585
AFP level (ng/mL)	49.8 (18–3000)	7.1 (4.8–1429.1)	0.149	235 (5.1–9950)	186 (4.1–458.0)	0.299
CA19-9 level (U/mL)	51.9 (39–325)	36.0 (14.5–165.6)	0.149	15 (2–63)	21 (3.1–82.0)	0.681
Tumor number, Single	3 (60.0)	5 (71.4)	1.000	7 (77.8)	5 (71.4)	1.000
Presence of micro-vascular invasion	3 (60.0)	1 (14.3)	0.222	5 (55.6)	2 (28.6)	0.358
Differentiation			0.773			1.000
Well	2 (40.0)	3 (42.9)		6 (66.7)	5 (71.4)	
Moderate	1 (20.0)	3 (42.9)		1 (11.1)	2 (28.6)	
Poor	2 (40.0)	1 (14.3)		2 (22.2)	0	
Presence of perineural invasion	1 (20.0)	0	0.417	1 (11.1)	0	
Within Milan criteria	2 (40.0)	7 (100.0)	0.045	4 (44.4)	7 (100.0)	0.034
Tumor stage			0.369			
I	1 (20.0)	4 (57.1)		3 (33.3)	4 (57.1)	0.615
II	3 (60.0)	3 (42.9)		6 (66.7)	3 (42.9)	
III	1 (20.0)	0		0	0	
Preoperative treatment	2 (40.0)	1 (14.3)	0.523	4 (44.4)	3 (42.9)	1.000
Postoperative adjuvant therapy	2 (40.0)	0	0.152	2 (22.2)	1 (14.3)	1.000

**Table 4:** Comparisons according to selection criteria in subgroups.

	ICC (n = 12)			CHC (n = 16)		
Variable	Within Milan Criteria (n = 9)	Beyond Milan Criteria (n = 3)	*p*-Value	Within Milan Criteria (n = 11)	Beyond Milan Criteria (n = 5)	*p*-Value
Age, years	49 (39–73)	51 (50–58)	0.516	54 (37–62)	52 (35–59)	0.733
Gender, Male	8 (88.9)	2 (66.7)	0.455	9 (81.8)	4 (80.0)	1.000
Presence of HBV infection	8 (88.9)	3 (100)	1.000	7 (63.6)	4 (80.0)	1.000
Presence of cirrhosis	7 (77.8)	2 (66.7)	1.000	9 (81.8)	3 (60.0)	0.547
AFP level (ng/mL)	19.5 (4.8–1429.1)	769.3 (18–3000)	0.209	186 (4.1–458)	485 (100–9950)	0.090
CA19-9 level (U/mL)	39.0 (14.5–165.6)	65.3 (51.9–325)	0.036	16.7 (2–82)	25.0 (10.3–63)	0.441
Maximum tumor size (cm)	2.5 (0.8–4.0)	10 (10–12)	0.015	3.00 (1.5–4.5)	6.50 (5–10)	0.002
Tumor number, Single	7 (77.8)	1 (33.3)	0.236	9 (81.8)	3 (60.0)	0.547
Presence of micro-vascular invasion	2 (22.2)	2 (66.7)	0.236	5 (45.5)	2 (40.0)	1.000
Differentiation			1.000			0.471
Well	4 (44.5)	1 (33.3)		8 (72.7)	3 (60.0)	
Moderate	3 (33.3)	1 (33.3)		3 (27.3)	1 (20.0)	
Poor	2 (22.2)	1 (33.3)		0	1 (20.0)	
Presence of perineural invasion	1 (11.1)	0	1.000	1 (9.1)	0	1.000
Tumor stage			0.159			1.000
I	5 (55.6)	0		5 (45.5)	2 (40.0)	
II	4 (44.4)	2 (66.7)		6 (54.5)	3 (60.0)	
III	0	1 (33.3)		0	0	
Preoperative treatment	2 (22.2)	1 (33.3)	1.000	4 (36.4)	3 (60.0)	0.596
Postoperative adjuvant therapy	1 (11.1)	1 (33.3)	0.455	1 (9.1)	2 (40.0)	0.214

## Data Availability

No new data were created, or where data is unavailable due to privacy or ethical restrictions.

## Supplementary Materials

The following supporting information can be downloaded at https://www.mdpi.com/article/10.3390/jcm14248857/s1. Figure S1: Survival outcomes according to tumor size in subgroups: (A) OS in patients with ICC over 3 cm versus less than 3cm, (B) RFS in patients with ICC over 3cm versus less than 3 cm, (C) OS in patients with CHC over 3cm versus less than 3 cm, (D) RFS in patients with CHC over 3 cm versus less than 3cm; Figure S2: Survival outcomes according to Milan Criteria in subgroups: (A) OS in ICC patients within Milan Criteria versus beyond Milan Criteria, (B) RFS in ICC patients within Milan Criteria versus beyond Milan Criteria, (C) OS in CHC patients within Milan Criteria versus beyond Milan Criteria, (D) RFS in CHC patients within Milan Criteria versus beyond Milan Criteria; Figure S3: Survival comparison by preoperative treatment: (A) OS in patients with presence of preoperative treatment versus absence of treatment, (B) RFS in patients with presence of preoperative treatment versus absence of treatment.
