# Supplementary material for: Outcomes of Liver Transplantation in Incidental Intrahepatic Cholangiocarcinoma and Combined Hepatocellular-Cholangiocarcinoma: An Exceptional Perspective from a Single-Center Experience
Source: J Clin Med. 2025 Dec 15;14(24):8857. doi: 10.3390/jcm14248857 (PMC12733566; doi:10.3390/jcm14248857)
Supplement: Supplementary file 1 [file jcm-14-08857-s001.zip › jcm-3972447-supplementary.docx]

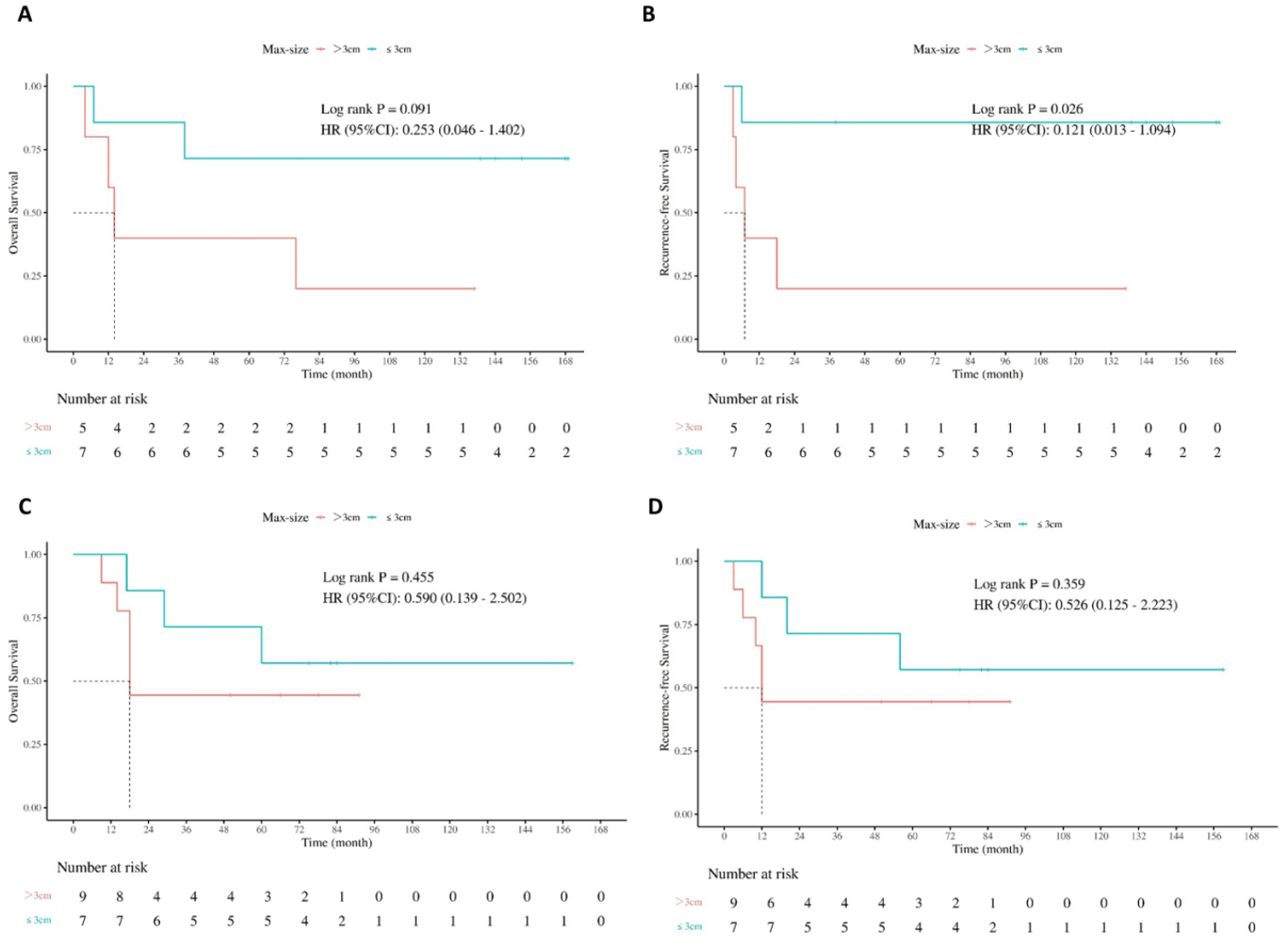
**Supplementary Figure S1.** Survival outcomes according to tumor size in subgroups: (A) OS in patients with ICC over 3cm versus less than 3cm, (B) RFS in patients with ICC over 3cm versus less than 3cm, (C) OS in patients with CHC over 3cm versus less than 3cm, (D) RFS in patients with CHC over 3cm versus less than 3cm.


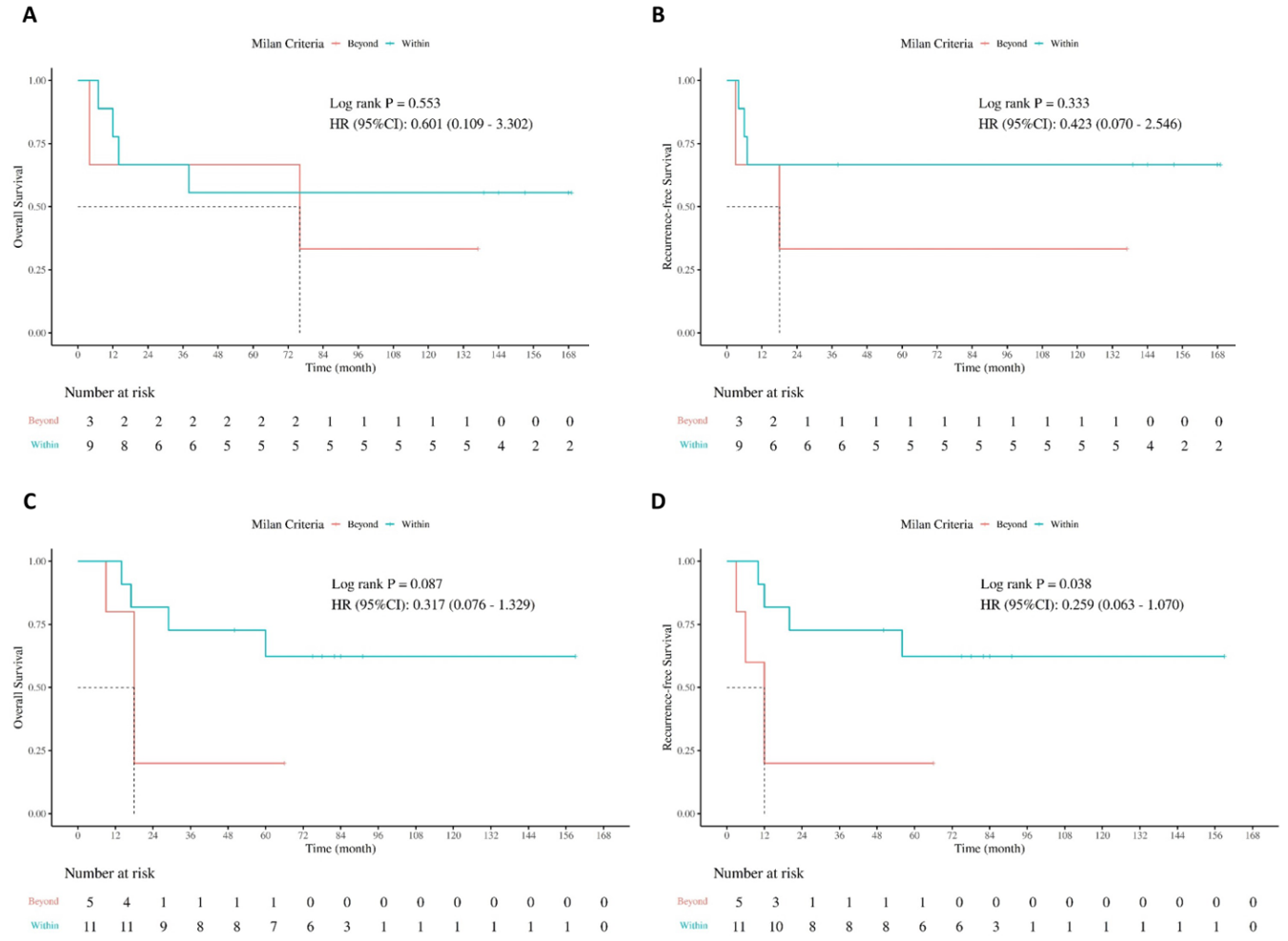


**Supplementary Figure S2.** Survival outcomes according to Milan Criteria in subgroups: (A) OS in ICC patients within Milan Criteria versus beyond Milan Criteria, (B) RFS in ICC patients within Milan Criteria versus beyond Milan Criteria, (C) OS in CHC patients within Milan Criteria versus beyond Milan Criteria, (D) RFS in CHC patients within Milan Criteria versus beyond Milan Criteria.


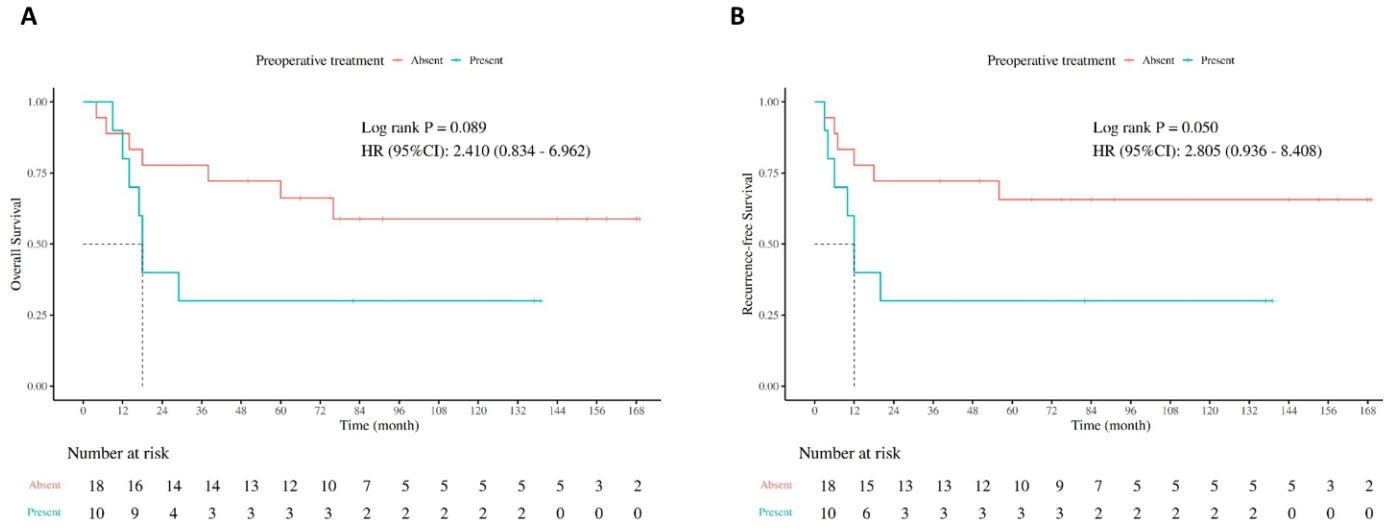


**Supplementary Figure S3.** Survival comparison by preoperative treatment: (A) OS in patients with presence of preoperative treatment versus absence of treatment, (B) RFS in patients with presence of preoperative treatment versus absence of treatment.
